# Effects of Resistance Training with Blood Flow Restriction on Explosive Power of Lower Limbs: A Systematic Review and Meta-Analysis

**DOI:** 10.5114/jhk/168308

**Published:** 2023-07-06

**Authors:** Xiaolin Wang, Xin-Min Qin, Shuyu Ji, Delong Dong

**Affiliations:** 1 Department of Sports Studies, Faculty of Educational Studies, University Putra Malaysia, Selangor, Malaysia.; 2 Department of Smart Health Science and Technology Convergence, Kangwon National University, Chuncheon, Korea.; 3 Department of Sport Science, Kangwon National University, Chuncheon, Korea.; 4 School of Teacher Education, Taizhou University, Zhejiang, China.; 5 Department of Physical Education, Ludong University, Shandong, China.

**Keywords:** explosive strength, occlusion training, cuff width, jumping, sprinting

## Abstract

The purpose of this systematic review and meta-analysis was to compare changes in explosive power between blood flow restriction training and traditional resistance training protocols. Searches of PubMed, Scopus, Web of Science, and OVID Medline were conducted for studies. Inclusion criteria were: (a) healthy people; (b) randomized controlled or controlled trials; (c) outcome measures of explosive performance (peak power, rate of force development, jump performance, sprint performance, etc.); (d) involving a comparison between blood flow restriction training and traditional resistance training. Quality assessment was conducted using the Physiotherapy Evidence Database (PEDro) scale. A total of 12 studies (262 subjects) were finally included for analysis. The PEDro scale score had a median of 5 of 10 points (range: 3–6 points). Significant small to moderate improvements were observed in blood flow restriction training [jump: standard mean difference (SMD) of 0.36 (95% CI: 0.02; 0.69); sprint: SMD of 0.54 (95% CI: 0.00; 1.07); power: SMD of 0.72 (95% CI: 0.17; 1.27)] when compared to traditional resistance training. The findings indicate that blood flow restriction training is more effective in improving explosive power of lower limbs compared to traditional resistance training in healthy people. In addition, blood flow restriction with a wide cuff (≥ 10 cm) during training improved explosive power better than with a narrow cuff or during the rest interval. Blood flow restriction training is very suitable for athletes in short competitive seasons and those who are not able to tolerate high loads (i.e., rehabilitators and the elderly).

## Introduction

Explosive power is the ability to exert great muscular strength in a very short time (usually within 100 ms) (Maffiuletti et al., 2016), which is vital for the sports population. It can help athletes win in competitive sports, prevent people from injury in sports, and even prevent the elderly from falling while walking (Granacher et al., 2008). Resistance training has long been viewed as the primary way to improve explosive power. However, training intensity is usually as high as 80% of an individual’s concentric 1-repetition maximum (1-RM) (Shinohara et al., 1997; Takarada, et al., 2000a). This high training intensity is not suitable for the elderly, untrained people, and short seasonal sports athletes, since it may lead to injuries. It would therefore seem logical that developing a more effective and efficient way to improve explosive power, in a relatively short period of time, would be essential for such populations.

Blood flow-restricted training (BFRT) may provide an alternative method to traditional high- intensity resistance training (RT). BFRT is low-intensity (20–30% of 1-repetition maximum) resistance training in combination with blood flow restriction. This training method has been shown to rapidly increase muscle hypertrophy and strength (Abe et al., 2005; Manimmanakorn et al., 2013; Yamanaka et al., 2012; Boyanmis and Akın 2022). Even walk training with restricted venous blood flow has been demonstrated to significantly improve leg-muscle strength and hypertrophy (Abe et al., 2006). Although the effects of BFRT on muscle hypertrophy and strength are well characterized, little is known about whether or not BFRT may improve explosive power.

The acute effects of post-activation potentiation in conjunction with blood flow restriction have been shown to improve sprint, jump and power performance (Doma et al., 2020; Hecht et al., 2016; Lindner et al., 2021), but chronic effects are still under debate. Cook et al. (2014) reported that maximal-sprint time and countermovement-jump power in rugby players were significantly improved after BFRT when compared with traditional resistance training (TRT) (*p* = 0.0162, 0.4% ± 0.3%; *p* = 0.0003, 1.8% ± 0.7%) (Cook et al., 2014). However, Scott et al. (2017) found through their research that there was no significant difference in the improvement of sprint and jump performance between BFRT and TRT. Similarly, through examining the effects of BFRT and TRT on the 30-m dash and standing jump, Abe et al. (2005) reported that BFRT significantly improved sprint performance more than TRT, but there was no large difference in jump performance (Liberati et al., 2009). There is at the present time no consensus on which training method is more beneficial for the improvement of explosive performance.

Therefore, the purpose of this meta-analysis was to investigate whether BFRT resulted in greater improvements in explosive power (i.e., peak power or the rate of force development, jump performance, sprint performance) compared with TRT.

## Methods

### Experimental Approach to the Problem

This systematic review was conducted according to the Cochrane Collaboration Guidebook and the criteria of Preferred Reporting Elements for Systematic Reviews and Meta-analyses (De Morton, 2009).

### Search Strategy

Articles published by August 12, 2022, were located using the electronic databases PubMed, SCOPUS, Web of Science, and OVID Medline. The following search syntax was used: “Blood flow restriction” OR “Blood flow occlusion” OR “restricted blood flow” OR “Kaatsu” AND “power” OR “jump” OR “sprint” OR “performance”. The lead author's personal libraries and gray literature sources (e.g., conference proceedings) were also examined. The systematic search process was conducted by X.-M.Q. and S.J. Any disagreement of the included/excluded study was resolved by the third author (X.W.).

### Selection Criteria

The inclusion criteria were as follows: (a) studies were randomized controlled trials or controlled trials; (b) the related indexes of explosive power (peak power, the rate of force development, jump performance, sprint performance, etc.) were measured before and after training intervention; (c) studies involved a comparison between resistance training with and without blood flow restriction. The excluded records (a) were not available in English; (b) included unhealthy or disabled people; (c) were cross-sectional studies; (d) were meta-analysis or review articles; (e) did not use BFR as the primary training program.

### Data Extraction

Data extraction from the included studies was independently performed by two authors (X.-M.Q. and S.J.). The following data were extracted: the first author’s name, publication year, sample size, subjects' age (years), sex, training protocol (e.g., exercises, sets, repetitions), period (days or weeks), frequency (sessions/week), cuff location, cuff pressure (mm Hg), cuff width (cm), duration of blood flow restriction and outcome indicators (details in the eligibility criteria section). In instances where information was unavailable for outcome indicators (e.g., mean or mean difference, standard deviation), lead authors were contacted for data. If no response was received, the study was excluded. Any disagreement in data extraction was resolved by the third author (X.W.).

### Risk of Bias of Studies

The Physiotherapy Evidence Database (PEDro) scale (Stojanović et al., 2017) was used to assess the risk of all included studies. There are 11 items in the PEDro checklist for a total of 10 points (item 1 is not rated). As in a similar previous plyometric training meta-analysis, literature quality was interpreted as “low quality” ( ≤ 3 points), “medium quality” (4–5 points), or “high quality” (6–10 points) (Hedges et al., 2014). Two reviewers (S.J. and D.D.) performed this rating independently. Any disagreement in rating was resolved by the third author (X.W.).

### Statistical Analyses

Effect sizes (ES) and 95% confidence intervals (CI) were calculated to compare the outcome measures of the included studies (Hedges et al., 2014). The mean difference and standard deviation of the outcome measures were used for calculating the effect sizes (Hedges’ *g*) due to baseline differences between the experimental and control groups. Calculated effect sizes were interpreted using the following scale: 0.2–0.5 = small, 0.5–0.8 = moderate, > 0.8 = large (Zlowodzki et al., 2007). We used the random-effects model to weight the standard errors (Borenstein et al., 2010) because the included studies had differences in populations, training intervention programs, outcome measures, and variables. The Review Manager program (RevMan; Version 5.4.; Copenhagen: The Nordic Cochrane Centre, The Cochrane Collaboration, 2014) was used to analyze the synthetic effects of BFRT and RT on explosive performance changes (i.e., jump, sprint, and power).

## Results

**Figure 1:**
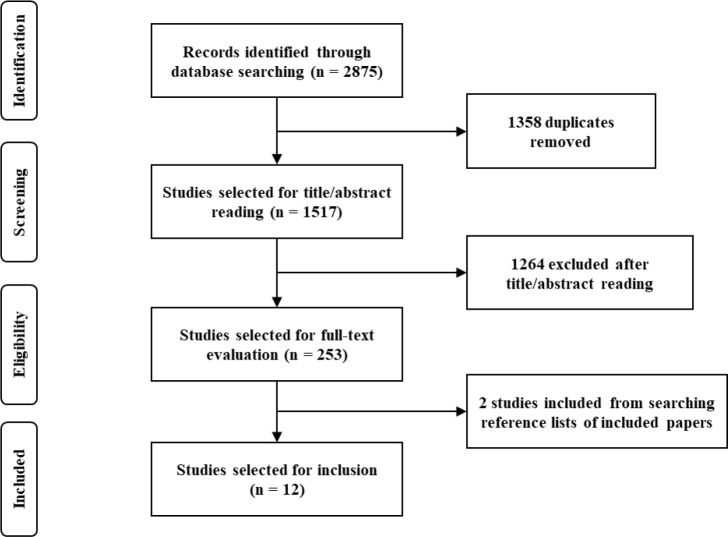
Flow-chart.

Figure 1 shows the selection process of the study literature. Through screening and exclusion, 12 studies were included in the meta-analysis. Eight studies provided jump data, six studies provided sprint data, and five studies provided muscular power data.

### Study Characteristics

The basic characteristics of participants, training programs, compression methods, and outcomes are displayed in Table 1. The included studies involved 262 participants with an age range of 13–34 years. Most of the subjects were young adults. Only one study included youth participants. In addition, the majority of study participants were athletes and physically active people. Only two studies included untrained people. The training load was 20–70% 1RM. The shortest training duration was 8 days and the longest was 10 weeks. The cuff location was the proximal part of the thigh, and the cuff pressure was 130–240 mm Hg (three studies applied 7/10 perceived pressure). The details are shown in Table 1.

**Table 1:** Study characteristics.

Studies	Subjects; N (BFRT, RT); Age	Training Protocol (sets × repetitions)	Training period, Frequency	BFR Duration, Cuff Location, Width, Pressure (cm, mm Hg)	Outcomes
Abe et al. (2005)	Male collegiate athletes; N = 9, 6; unclear	Squats and leg curls: 3 sets × 15 reps at 20% 1RM	8 days,2 sessions/day	Throughout RT, proximal part of the thigh, n,160‒240	30-m dash↑0.6% ┼; jump ↑2%
Behringer et al. (2017)	Male sport students; N = 12, 12; 19‒27	6 consecutive 100-m sprints at an intensity of 60‒70% of best sprinting performance	6 weeks, 2 sessions/week	Throughout RT, proximal part of the thigh, 13, 7/10 PP	RFD leg press↑24.9% ┼; sprint time↑3% ┼
Cook et al. (2014)	Rugby players; N = 10, 10; 21.5 (1.4)	Bench press, leg squat, and pull-ups: 5 sets × 5 reps at 70% 1RM	3 weeks, 3 sessions/week	During exercise, proximal part of the thigh, 10.5, 180	Sprint time↓0.4% ┼; CMJ power↑1.8% ┼
de Oliveira et al. (2016)	Young adults; N = 10, 7; 23.8 (4)	Interval-training: 2 sets × (5‒8) reps at 30% and 100% of maximal power output	4 weeks, 3 sessions/week	During exercise, proximal part of the thigh, 18, 140‒200	Isometric knee extension peak power↑9.8% ┼
Horiuchi et al. (2018)	Young males; N = 10, 10; 21‒25	Vertical jumps: 5 sets × 10 reps	4 weeks, 4 sessions/week	During rest intervals, proximal part of the thigh, 11, 200	CMJ↓5.5%
Sa et al. (2020)	Male soccer players; N = 10, 9; 15‒17	45‒65 min soccer training: soccer drills, small-sided games, plyometric training, continuous running	6 weeks, 3 sessions/week	Throughout RT, proximal part of the thigh, 4, 160‒210	CMJ↑1.7%
Madarame et al. (2011)	Physically active men; N = 8, 7; 18‒22	Horizontal squat machine: 4 sets × (15‒30) reps at 30% 1RM	10 weeks, 2 sessions/week	Throughout RT, proximal part of the thigh, n, 200‒250	CMJ↑4.4%
Manimmanakorn et al. (2013)	Female netball athletes; N = 10, 10; 20.2 (3.3)	Bilateral knee flexion and extension: 3 sets to failure at 20% 1RM	5 weeks, 3 sessions/week	Throughout RT, proximal part of the thigh, n, 160‒230	sprint time↑2.9%; vertical jump↑4.9%
Scott et al. (2017)	Male soccer players; N = 10, 8; 19.8 (1.5)	Jumps and low-load squats: 4 sets × (15‒30) reps at 20‒30% 1RM	5 weeks, 3 sessions/week	Throughout RT, proximal part of the thigh, 7.5,7/10 PP	Sprint time↑0%; CMJ ↑0%
Amani-Shalamzari et al. (2020)	Male futsal players; N = 6, 6; 23 (2)	3-a-side SSG play	3 weeks, 10 sessions in total	During exercise, proximal part of the thigh, 13, 110‒150% LSBP	30-s Wingate test peak power↑7.9%
Taylor et al. (2016)	Healthy trained men; N = 10, 10; 20‒34	30-s maximal sprint cycling bouts: 4‒7 sets	4 weeks, 2 sessions/week	During rest intervals, proximal part of the thigh, n, 130	Sprint peak power↑0.1%
Yang et al. (2022)	Trampoline gymnasts; N = 7, 8; 13.9 (0.4)	Back squats, front squats at 25‒35% 3RM, squat jump at 20‒30% BW, CMJ at 20 kg, hurdle jumps and drop jumps at BW	10 weeks, 2 sessions/week	Throughout RT, proximal part of the thigh, 7.62, 7/10 PP	CMJ ↑4.8% ┼

Note: ┼ = statistically significant between group difference (BFRT vs. TRT). 1RM = one repetition maximum, BW = body weight, CMJ = countermovement jump, LSBP = leg individual's systolic blood pressure, n = not given, PP = perceived pressure, RT = resistance training.

### Methodological Quality of Included Studies

Among the included studies, all studies achieved 4–5 points (moderate quality) except one study (3 points). The PEDro scale score had a median of 5 of 10 points across studies (Table 2).

**Table 2:** Physiotherapy evidence database (PEDro) scale ratings.

References	Items*											Total (from a possible maximal of 10)
	1	2	3	4	5	6	7	8	9	10	11	
Abe et al. (2005)	1	1	0	1	0	0	0	1	1	1	1	6
Behringer et al. (2016)	1	1	0	0	0	0	0	1	1	1	1	5
Cook et al. (2014)	1	0	0	0	0	0	0	1	1	1	1	4
de Oliveira et al. (2015)	1	0	0	0	0	0	0	1	1	1	1	4
Horiuchi et al. (2018)	1	0	0	0	0	0	0	1	1	1	1	4
Kakhak et al. (2020)	1	1	0	1	0	0	0	1	1	1	1	6
Madarame et al. (2011)	1	0	0	0	0	0	0	0	1	1	1	3
Manimmanakor et al. (2013)	1	1	0	0	0	0	0	1	1	1	1	5
Scott et al. (2016)	1	1	0	1	0	0	0	1	1	1	1	6
Shalamzari et al. (2020)	1	1	0	0	0	0	0	1	1	1	1	5
Taylor et al. (2016)	1	1	0	1	0	0	0	1	1	1	1	6
Yang et al. (2022)	1	1	0	0	0	0	0	1	1	1	1	5
												Median score = 5

Note: * a detailed explanation for each PEDro scale item can be accessed at https://pedro.org.au/wp-content/uploads/PEDro_scale.pdf (access for this review: August 26, 2022).

### Synthesis of Results

**Figure 2:**
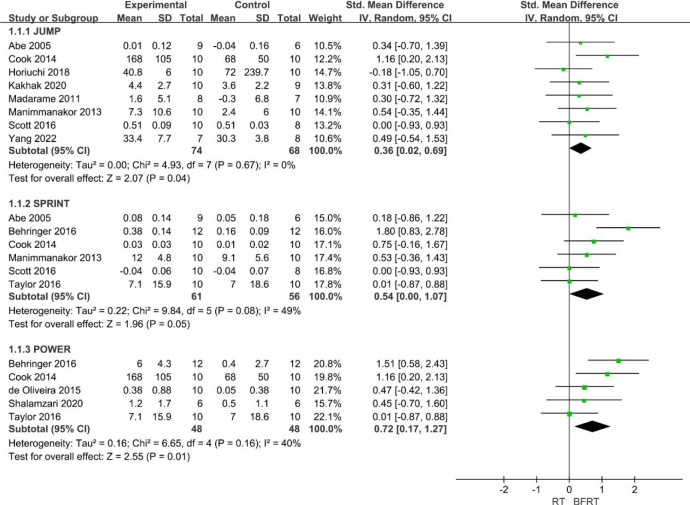
Effects of BFRT on power.

Combined data from eight studies revealed a small, significant effect in favor of BFRT for improvements in jump performance (*p* < 0.05, SMD = 0.36 [0.02, 0.69] at 95% CI) (Figure 3). A moderate, significant effect was found in favour of BFRT for improvements in sprint performance (6 studies, *p* = 0.05, SMD = 0.54 [0.00, 1.07] at 95% CI). A moderate, significant effect was found in favour of BFRT for improvements in power performance (5 studies, *p* = 0.01, SMD = 0.54 [0.17, 1.27] at 95% CI). Small to medium levels of statistical heterogeneity were presented and the chi-square test was not significant (I^2^ = 0–49%, *p* = 0.08–0.67) (Figure 2).

## Discussion

To the best of our knowledge, this is the first review that examined the effects of blood-flow restricted training (BFRT) on muscular explosive power when compared to those of traditional resistance training (TRT). The main finding was that BFRT induced greater improvements in explosive performance (i.e., jump, sprint, and power) than TRT. This finding underlines the potential of BFRT as a more effective and efficient low-intensity alternative to TRT for promoting gains in muscular explosive power in healthy populations.

The greater improvements in explosive power with BFRT vs. TRT may be related to neuromuscular adaptation induced by blood flow restriction.

The first is the promotion of the neuromuscular drive. Studies by Moritani et al. (1992) and Takarada et al. (2000b) showed that BFRT induced earlier and greater fast muscle fiber recruitment due to insufficient oxygen supply to slow muscle fibers. The second is the improvement in muscle oxygen uptake capacity and utilization of phosphocreatine (de Mendonca et al., 2022; Lindner et al., 2021; Paradis-Deschênes et al., 2016). In addition, it has been observed that the benefits of BFRT may, in part, be related to the muscle protein synthesis and a concomitant decrease in the mRNA gene expression of MURF-1, atrogin, and myostatin (Gundermann et al., 2012; Laurentino et al., 2012; Manini et al., 2011). These neuromuscular adaptations are particularly important for the improvement of explosive power.

Based on the present meta-analysis results, we found that BFRT had greater training specificity than TRT. For example, sprint performance changed the most (3%, *p* < 0.05) in the study by Behringer et al. (2017) due to the use of sprint training. A study by Yang et al. (2022) involved a variety of jumping exercises, resulting in a significant improvement in jump performance (4.8%, *p* < 0.05). However, another study which implemented jump training showed a reduced jump performance (Horiuchi et al., 2018). This may be due to the uniformity of the training schedule (only vertical jump, fixed training volume and load). Thus, we should not only consider the training specificity, but also pay attention to the rationality of the training plan when applying BFRT.

Another interesting finding was that different types of blood flow restriction (BFR) had different effects on explosive power, especially BFR duration and cuff width. A study by Jessee et al. (2016) found that wide cuffs reached arterial occlusion pressure (AOP) at a lower value than narrow cuffs. This means that wide cuffs induce a greater flow restriction than narrow cuffs at the same inflation pressure, which results in better effectiveness of resistance training (Loenneke et al., 2013; Rossow et al., 2012; Wilk et al., 2020). The results of the present review are consistent with the argument. Except for the study by Horiuchi et al. (2018), due to compression used only during the rest interval (ES = −0.18), BFRT with wide cuffs (≥ 10 cm) has a more significant effect on explosive power than with narrow cuffs (< 10 cm, 0.45−1.51 vs. 0−0.49). Although it has certain advantages, the wide cuff restricts movement and may lead to safety problems at high pressure. Therefore, it is recommended to set the inflation pressure according to the cuff width and individual value of arterial occlusion pressure (% AOP) (Patterson et al., 2019). In terms of BFR duration, Wilk et al. (2020a) demonstrated that continuous and intermittent BFRT had similar effects on movement velocity during the bench press exercise. This is consistent with the results of the present review. We found that BFR throughout resistance training and only during exercise had similar effects on explosive power (0.3−1.8 vs. 0.45−1.16), except for the study by Scott et al. (2017) (ES = 0) due to the narrow cuff. In addition, we also found that the influence of BFR only during the rest interval on explosive power (ES = −0.18−0.01) was lower than that of BFR throughout resistance training and only during exercise. This is probably because the combination of exercise and BFR induces greater physiological adaptation than BFR only during the rest period. Therefore, BFR with a wide cuff during training may be a better choice for improving explosive power.

The BFRT program plays a vital role in the enhancement of explosive power. Firstly, when selecting a training programs, attention should be paid to training specificity and variety. Secondly, the training load should be approximately 20–35% of one-repetition maximum. Although a study with 70% 1 RM load by Cook et al. (2014) showed an increase in explosive power, another study by de Oliveira et al. (2016) demonstrated a decrease in explosive power at such intensity. Apart from the uncertainty of the effect on explosive power, BFRT with a high load may cause safety problems (cardiovascular system, muscle damage, oxidative stress etc.) (Loenneke et al., 2011). Thirdly, the mode of the wide cuff (≥ 10 cm) at the most proximal part of the thigh and BFR during training may be a better choice for improving explosive power of lower limbs.

In addition, the training period in most studies of this review was 3‒4 weeks. The study by Abe et al. (2005) even showed an improvement in explosive power after 8 days of training. These results indicate that BFRT has a faster effect on the improvement in explosive power than TRT. The fast effect and low load characteristics of BFRT make it suitable for athletes in a short competitive season or those who are not able to tolerate high loads (e.g., rehabilitation patients and the elderly).

The main limitation of the present meta-analysis is the small number of included studies. Although the role of BFRT in improvements in muscle strength has received a lot of attention in recent years (Luebbers et al., 2014; Maciel Batista et al., 2020; Yamanaka et al., 2012), only few studies have focused on BFRT aimed at the improvement in explosive power. Considering the importance of improving explosive power under low load training conditions for athletes, rehabilitators or the elderly, future research analyzing the effects of BFRT on explosive power is thus warranted. This may provide valuable information for coaches, physiotherapists, and scientists for managing training programs.

## Practical Implications

The present meta-analysis shows that blood flow-restricted training (BFRT) induces greater improvements in explosive strength of lower limbs (i.e., jump, sprint, and power) compared with traditional resistance training (TRT). It seems that a BFRT program consisting of 2–4 resistance exercises at 20–35% 1RM per week, with a wide cuff (≥ 10 cm) and individual inflation pressure (according to arterial occlusion pressure) at the most proximal part of the thigh, blood flow restriction during training, and lasting 3–4 weeks is an appropriate strategy for improving explosive power of lower limbs. This may help athletes achieve optimum explosive performance in a short competitive season, and help rehabilitators or the elderly achieve optimum exercise effects with a low load.
